# Bardet-Biedl syndrome improved diagnosis criteria and management: Inter European Reference Networks consensus statement and recommendations

**DOI:** 10.1038/s41431-024-01634-7

**Published:** 2024-07-31

**Authors:** Hélène Dollfus, Marc R. Lilien, Pietro Maffei, Alain Verloes, Jean Muller, Giacomo M. Bacci, Metin Cetiner, Erica L. T. van den Akker, Monika Grudzinska Pechhacker, Francesco Testa, Didier Lacombe, Marijn F. Stokman, Francesca Simonelli, Aurélie Gouronc, Amélie Gavard, Mieke M. van Haelst, Jens Koenig, Sylvie Rossignol, Carsten Bergmann, Miriam Zacchia, Bart P. Leroy, Héléna Mosbah, Albertien M. Van Eerde, Djalila Mekahli, Aude Servais, Christine Poitou, Diana Valverde

**Affiliations:** 1 ERN-EYE Centre de Référence Pour les Affections Rares en Génétique Ophtalmologique (CRMR CARGO), Institut de Génétique Médicale d’Alsace (IGMA), FSMR SENSGENE, Hôpitaux Universitaires de Strasbourg, Strasbourg, France; 2 Université de Strasbourg, UMRS_1112, Strasbourg, France; 3 ERKNet Wilhelmina Children’s Hospital, University Medical Center, Utrecht, The Netherlands; 4 Endo-ERN Department of Medicine (DIMED), 3rd Medical Clinic, Padua University, Padua, Italy; 5 ERN-ITHACA Department of Genetics, AP-HP - Université de Paris; INSERM UMR 1141 “NeuroDiderot”, Hôpital Robert Debré, Paris, France; 6 Laboratoires de Diagnostic Génétique, Hôpitaux Universitaires de Strasbourg, Strasbourg, France; 7 Unité Fonctionnelle de Bioinformatique Médicale Appliquée au Diagnostic (UF7363), Hôpitaux Universitaires de Strasbourg, Université de Strasbourg, UMRS_1112, Strasbourg, France; 8 ERN-EYE Pediatric Ophthalmology Unit, Meyer Children’s Hospital IRCCS, University of Florence, Florence, Italy; 9 ERKNet Children’s Hospital, Pediatrics II, University of Essen, Essen, Germany; 10 Endo-ERN Obesity Center CGG, Erasmus MC, University Medical Center Rotterdam, Division of Endocrinology, Department of Pediatrics, Erasmus MC-Sophia, University Medical Center Rotterdam, Rotterdam, The Netherlands; 11 ERN-EYE Coordination Center, Hôpitaux Universitaires de Strasbourg, Strasbourg, France; 12 ERN-EYE Eye Clinic, Multidisciplinary Department of Medical, Surgical and Dental Sciences, University of Campania Luigi Vanvitelli, Naples, Italy; 13 ERN-ITHACA Department of Medical Genetics, CHU Bordeaux, INSERM Unit_1211, Laboratory “Rare Diseases: Genetics and Metabolism”, University of Bordeaux, Bordeaux, France; 14 ERKNet Department of Human Genetics, Radboud University Medical Centre, Nijmegen, The Netherlands; 15 ERN-ITHACA Department of Human Genetics, Section Clinical Genetics, Amsterdam UMC location University of Amsterdam, Amsterdam, The Netherlands; 16 ERKNet University Children’s Hospital Muenster, Muenster, NRW Germany; 17 Endo-ERN Département de Pédiatrie, Hôpitaux Universitaires de Strasbourg, Strasbourg, France; 18 Department of Medicine IV, Faculty of Medicine, Medical Center, University of Freiburg, Freiburg, Germany; 19 Medizinische Genetik Mainz, Limbach Genetics, Mainz, Germany; 20 ERKNet Division of Nephrology, Department of Translational Medical Sciences, University of Campania “L. Vanvitelli”, Naples, Italy; 21 ERN-EYE Department of Ophthalmology & Department of Head & Skin, Ghent University Hospital and Ghent University, Ghent, Belgium; 22 Center for Cellular and Molecular Therapeutics and Division of Ophthalmology, Children’s Hospital of Philadelphia, Philadelphia, PA USA; 23 Endo-ERN Department of Endocrinology, Diabetology & Nutrition, University Hospital of Poitiers, Poitiers, France; 24 ERKNet Department of Genetics, University Medical Center Utrecht, Utrecht, The Netherlands; 25 ERKNet PKD Research Group, Department of Cellular and Molecular Medicine, KU Leuven, Leuven, Belgium; 26 Department of Pediatric Nephrology, University Hospitals Leuven, Leuven, Belgium; 27 ERKNet Department of Kidney and Metabolic Diseases, Transplantation and Clinical Immunology, Necker Hospital, AP-HP, Centre of Reference for the French Nationwide MARHEANetwork (CNR-MARHEA), Paris, France; 28 Inserm U1163, Imagine Institute, Paris, France; 29 Endo-ERN Centre de Référence pour les obésités rares (CRMR PRADORT), Assistance Publique Hôpitaux de Paris, Pitié-Salpêtrière Hospital, Sorbonne Université, INSERM, Nutrition & Obesities: Systemic Approaches Research Group (NutriOmics), Paris, France; 30 CINBIO, Universidad de Vigo, Grupo de Investigación en Enfermedades Raras, Instituto de Investigación Sanitaria Galicia Sur (IIS Galicia Sur), Vigo, Spain

**Keywords:** Genetic testing, Therapeutics

## Abstract

Four European Reference Networks (ERN-EYE, ERKNet, Endo-ERN, ERN-ITHACA) have teamed up to establish a consensus statement and recommendations for Bardet-Biedl syndrome (BBS). BBS is an autosomal recessive ciliopathy with at least 26 genes identified to date. The clinical manifestations are pleiotropic, can be observed in utero and will progress with age. Genetic testing has progressively improved in the last years prompting for a revision of the diagnostic criteria taking into account clinical Primary and Secondary features, as well as positive or negative molecular diagnosis. This consensus statement also emphasizes on initial diagnosis, monitoring and lifelong follow-up, and symptomatic care that can be provided to patients and family members according to the involved care professionals. For paediatricians, developmental anomalies can be at the forefront for diagnosis (such as polydactyly) but can require specific care, such as for associated neuro developmental disorders. For ophthalmology, the early onset retinal degeneration requires ad hoc functional and imaging technologies and specific care for severe visual impairment. For endocrinology, among other manifestations, early onset obesity and its complications has benefited from better evaluation of eating behaviour problems, improved lifestyle programs, and from novel pharmacological therapies. Kidney and urinary track involvements warrants lifespan attention, as chronic kidney failure can occur and early management might improve outcome. This consensus recommends revised diagnostic criteria for BBS that will ensure certainty of diagnosis, giving robust grounds for genetic counselling as well as in the perspective of future trials for innovative therapies.

## Introduction

Bardet-Biedl syndrome (BBS) (OMIM#209901; ORPHA: 110) is an emblematic ciliopathy affecting multiple organs and requiring multidisciplinary care from early life on. BBS is a highly disabling condition because of early-onset retinal degeneration, early morbid obesity and regular kidney involvement. Many other clinical manifestations can occur, such as polydactyly, neurodevelopmental disorders or various malformations. Prevalence is estimated to be ~ 1:160,000 [1] increasing to ~ 1:15,000 [2] in several isolated communities. Intra and inter familial variable expressivity is reported. Inheritance is autosomal recessive, with at least 26 BBS genes identified [3]. Triallelic inheritance (three variations in two BBS genes) has been proposed but is highly disputed. Second-order genetic modifiers have now been proposed as a cause of intra-familial variability in this disease [4]. Much of the pathogenesis is due to primary cilia dysfunction, as *BBS* genes are implied in ciliary pathways with multiple biological roles, thus explaining the pleiotropic clinical manifestations. Early clinical and genetic diagnosis, lifetime medical care and monitoring are key for improving the medical status, quality of life and life expectancy. This clinical consensus statement provides guidance for: BBS diagnosis with updated diagnostic criteria taking into account genetic testing; clinical initial evaluation; follow-up monitoring; recommendations to patient, family and caregivers. The current evidence was generated by multidisciplinary expertise provided by four European Reference Networks (ERNs) [5].

## Methods

This Clinical Consensus Statement (CCS) has been developed by a European group of expert physicians, geneticists, allied healthcare professionals and patient support groups with a common aim to support equitable care by establishing a consensus around the standard of care for all patients with BBS. The CCS executive group consisted of expert representatives from a range of professional groups including paediatric and adult ophthalmologists, paediatric and adult nephrologists, paediatric and adult endocrinologists, geneticists and patient support group representatives. The clinical experts are members of four European Reference Networks (ERN-EYE [6], ERKNet [7], Endo-ERN [8], ERN-ITHACA [9]). The experts worked in groups devoted to a specific specialty (Ophthalmology, Nephrology, Endocrinology, Developmental Anomalies and Genetics) and designated a lead. Each group formulated the clinical questions to be addressed by the executive group and the keywords to be used in the literature review.

Each group carried out a systematic literature review on BBS over the last 45 years until July 2022, using Medline, Embase and PubMed. Relevant published papers considered by the group members as important were included. Each group met by teleconference and corresponded by email on a regular basis throughout the duration of the CCS elaboration. They developed recommendations that were submitted to a vote using the Delphi method. The voting was performed anonymously. Participating experts were given five voting options, from 100% (full agreement) to 0% (complete disagreement). The average percentage was calculated for each statement. Anything with an approval average above 75% was considered to be accepted, statements with an average included between 75% and 25% went through a revision, before being send back to voting. Any statement with an average below 25% was dismissed. In addition, we formed a panel of experts including each group leader and one additional member of each group. This panel met in-person in September 2022 to elaborate new diagnostic criteria for BBS.

## Results

### Updated BBS diagnostic criteria

To date, the BBS diagnostic criteria used in clinical practice are based only on clinical features as the genetic landscape has progressively emerged [10]. Improved molecular diagnosis yield and natural history knowledge prompted CCS executive group to update the BBS diagnosis criteria. These new criteria are based on the age of the patient (in utero, childhood, adolescence and adulthood) and, most importantly, take into account molecular diagnosis available in most countries (Table 1). As genetic testing is not always accessible or fully completed, clinical criteria may still be used but with more stringent requirements. Clinical criteria have been split into two categories: “Primary criteria” defined as highly penetrant clinical manifestations, and “Secondary criteria” defined as BBS well-known features, however less frequent and/or less stringent for the diagnosis. Additional clinical manifestations, also mentioned in this statement, can occur as early manifestations or as complications of primary criteria. These numerous features were not added in order to simplify the use of these criteria in clinical practice. The updated criteria can provide “High level of evidence diagnosis” and therefore can be used for inclusion in future clinical trials, BBS specific prescriptions and genetic counselling. “Moderate levels of evidence diagnosis” is of utility for initial and follow-up clinical monitoring.

**Table 1:**
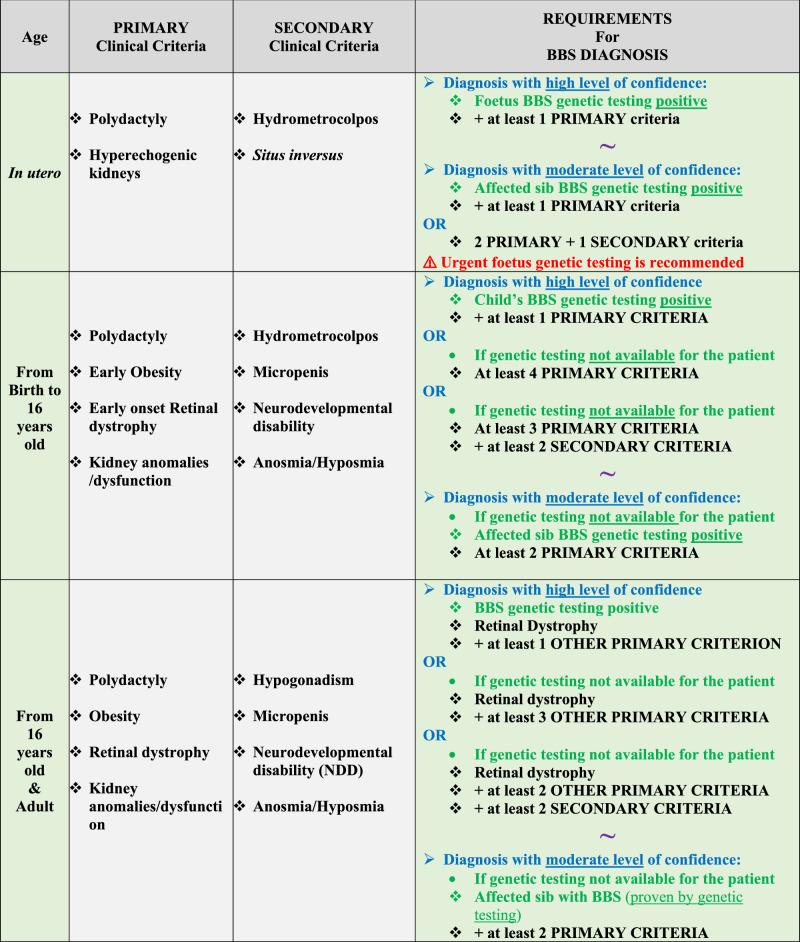
Revised Diagnosis criteria for BBS taking into account molecular diagnosis.

Genetic testing positive is defined as proven molecular diagnosis by Biallelic class 4-5 pathogenic variant(s) in a confirmed BBS gene (see chapter).

Genetic test is confirmed.

Genetic test is not confirmed.

❖ Clinical criteria.

### Genetic testing for BBS diagnosis: current status

BBS is a fully penetrant autosomal recessive condition, with variable expressivity. Genetic testing is of utility for diagnosis (Table 1), genetic counselling, as well as for inclusion in trials or specific prescriptions. Definitive BBS molecular diagnosis requires identification of pathogenic or likely pathogenic bi-allelic variants (class 4/5 of the American College of Medical Genetic and Genomics (ACMG) variant classification [11]) in a given BBS gene [12]. Currently, the total mutational load, taking into account all BBS genes and modifier loci, together with possible epistatic effects and environmental factors, is thought to contribute to the clinical variability of BBS patients but is not considered in routine diagnosis [12].

At least 26 genes are reported [3], most well confirmed, but a subset still has limited evidence and needs further replication to date (Table 2). Four categories of BBS genes are described: BBSome members, chaperonin-like members, Intra Flagellar Transport (IFT) genes and others. The detection rate of pathogenic variants in patients that fulfil the clinical diagnostic criteria for BBS is high (> 90%). About 40–50% of pathogenic variants in Europe are identified in *BBS1* and *BBS10*. Two common founder variants are well recognized: 1) *BBS1* exon 12: c.1169 T > G p.(Met390Arg) and 2) *BBS10* exon 2c.271dupT p.(Cys91Leufs*5). While the common *BBS1* variant is usually absent in non-European descending populations, the *BBS10* common variant can be found worldwide [3]. Except for the two mentioned changes and specific population founder variants, most variants are private and many are truncating with straightforward categorization as class 4 or 5 variants. The detection rate also depends on the method used: NGS-based approaches (panel, Whole Exome and Whole Genome Sequencing (WGS)) offer further advantages compared to conventional Sanger sequencing such as the possibility of detecting copy number variants. To date, it is recommended to use panels (with all BBS genes and main differential diagnosis such as Alström syndrome (ALMS, OMIM#203800, ORPHA: 64) and other overlapping ciliopathies) in first intention for diagnostic purposes. Segregation analysis with the parents DNA is always highly recommended especially: 1) for homozygous variants to rule out a masked deletion on one of the parental alleles or a uniparental disomy [13] and; 2) for compound heterozygotes to confirm the bi-allelic status (NB: de novo variants occur in rare cases) [13]. Partners of healthy heterozygous carriers (usually siblings) can be tested to evaluate the recurrence risk, especially in the context of *BBS1* and *BBS10* common variants or if they belong to a population with a founder variation. When the genetic testing confirms bi-allelic pathogenic status, according to the facilities accessible and regulation in a given country, couples can opt for prenatal genetic testing or preimplantation genetic testing. Non-invasive genetic diagnosis via cell free foetal DNA is not common but available in some European centres.

**Table 2:** List of the genes related to Bardet-Biedl syndrome (at the date of 08/2023).

Gene symbol	BBS	Gene synonyms	Gene category	OMIM	Phenotype	Nb variations (Class 4/5) reported	Status	Reference sequence (RefSeq)	Chromosome	Total nb exons	CDS length (nt)	Protein length (AA)
*BBS1*	*BBS1*		BBSome	209900	BBS, RP	> 180	Core	NM_024649.4	11	17	1782	593
*BBS2*	*BBS2*	*BBS2L1*	BBSome	606151	BBS, RP	> 140	Core	NM_031885.3	16	17	2166	721
*ARL6*	*BBS3*	*RP55*	Other	608845	BBS, RP	> 30	Core	NM_032146.4	3	11	561	186
*BBS4*	*BBS4*		BBSome	600374	BBS	> 70	Core	NM_033028.4	15	18	1560	519
*BBS5*	*BBS5*		BBSome	603650	BBS	> 40	Core	NM_152384.2	2	17	1026	341
*MKKS*	*BBS6*		Chaperonin-like	604896	BBS, RP+polydactyly	> 70	Core	NM_018848.3	20	7	1713	570
*BBS7*	*BBS7*		BBSome	607590	BBS	> 60	Core	NM_176824.2	4	20	2148	715
*TTC8*	*BBS8*	*RP51*	BBSome	608132	BBS, RP	> 20	Core	NM_144596.2	14	16	1518	515
*BBS9*	*BBS9*	*PTHB1*	BBSome	607968	BBS, RP	> 60	Core	NM_198428.2	7	24	2559	887
*BBS10*	*BBS10*		Chaperonin-like	610148	BBS, RP	> 180	Core	NM_024685.3	12	2	2172	723
*TRIM32*	*BBS11*	*HT2A, TATIP*	Other	602290	BBS, LGMD	1 paper reporting a single BBS case (homozygous missense), many cases of LGMD	Questioned	NM_012210.3	9	2	1962	653
*BBS12*	*BBS12*		Chaperonin-like	610683	BBS	> 100	Core	NM_152618.2	4	3	2133	710
*MKS1*	*BBS13*	*FLJ20345, POC12, BBS13*	Other	609883	BBS, MKS	Mostly truncating variants in MKS patients. Few missenses. No clear association with BBS (clinical details and variant missing)	Need replication	NM_017777.3	17	20	1680	559
*CEP290*	*BBS14*	*NPHP6, CEP290, MKS4, JBTS5, SLSN6*	Other	610142	BBS, LCA, MKS, NPHP, JBTS, SLNS	3 truncating variants in 2 families with corresponding phenotype (2 papers)	Need replication	NM_025114.3	12	55	7440	2479
*WDPCP*	*BBS15*	*fritz, hFrtz, CPLANE5*	Other	613580	BBS, RP, MKS, OFD	1 splice variant in 1 family	Need replication	NM_015910.5	2	19	2469	746
*SDCCAG8*	*BBS16*	*CCCAP, NPHP10, NY-CO-8, SLSN7*	Other	613524	BBS, NPHP, SLSN	> 10	Core	NM_006642.3	1	21	2141	713
*LZTFL1*	*BBS17*		Other	606568	BBS	3 variants (2 truncating + 1 missense). 3 patients in 2 families.	Need replication	NM_020347.3	3	10	2360	299
*BBIP1*	*BBS18*	*BBIP10*	BBSome	613605	BBS	2 families + functional data.	Core Need replication	NM_001195304.1	10	5	312	103
*IFT27*	*BBS19*	*RAYL*	IFT	615870	BBS	3 families + functional data.	Core	NM_001177701.1	22	7	1104	186
*IFT172*	*BBS20*	*NPHP17*	IFT	607386	BBS, RP, SRTD	Truncating variants + missenses, 10 families (personal communication)	Core	NM_015662.2	2	48	5247	1749
*CFAP48*	*BBS21*	*FLJ30600, CORD16, RP64, FAP418, MOT25, C8orf37*	Other	614477	BBS, RP	1 truncating variant in 1 family	Need replication	NM_177965.3	8	6	624	207
*IFT74*	*BBS22*	*CCDC2*	IFT	608040	BBS, JBTS	Truncating variants, 3 families + functional data	Core	NM_025203.3	9	20	1803	600
*CEP19*	*BBS23*	*C3orf34*	Other	615586	BBS, Morbid Obesity	1 truncating variant in 1 family	Need replication	NM_032818.4	3	3	504	168
*SCAPER*	*BBS24*	*ZNF291*	Other	611611	BBS, RP + /-ID, RP	3 reports and non-supporting BBS phenotype	Need replication Questioned	NM_001353009.1	15	33	4211	1406
*CEP164*	*BBS25*	*NPHP15*	Other	614848	BBS, NPHP, JBTS	1 truncating variant in 1 family	Need replication	NM_014956.4	11	33	4383	1460
*SCLT1*	*BBS26*		Other	611399	BBS, SLSN, OFD	2 splice variants (one in frame deletion) in 2 families	Need replication	NM_144643.4	4	21	2067	689
*NPHP1*		*NPH1, JBTS4, SLSN1*		607100	NPHP		Modifier	NM_000272.3	2	20	2202	733
*CCDC28B*		*MGC120, RP4-622L5.5*		610162			Modifier					
*TMEM67*		*MKS3, MGC26979, JBTS6, NPHP11*		609884	MKS, NPHP		Modifier					
*TTC21B*		*FLJ11457, JBTS11, NPHP12,IFT139B, THM1, FAP60, FLA17 IFT139*		612014			Modifier					
	Total									446	56934	18261

The publication references linked to the genes can be found with the OMIM number. The official genes names and their synonyms (HUGO Gene Nomenclature Committee) are given together with other annotations. Status of the gene regarding the syndrome has been assessed into several categories including gene undoubtedly associated with BBS (“Core”), gene with good evidence but requires confirmation (“Need replication”) and gene with some evidence but of low impact (“Questioned”). *BBS* Bardet-Biedl Syndrome, *CDS* Coding sequence, *ID* Intellectual Disability, *JBTS* Joubert Syndrome, *LCA* Leber Congenital Amaurosis, *LGMD* Limb Girdle Muscular Dystrophy, *MKS* Meckel Syndrome, *NPHP* Nephronophtisis, *OFD* Oro Facio Digital syndrome, *RP* Retinitis Pigmentosa, *SLNS* Senior-Løken Syndrome, *SRTD* Short-Rib Thoracic Dysplasia.

Clear-cut genotype-phenotype correlations remain difficult to implement in clinical practice because the studies are often based on limited numbers of patients.

The first replicated genotype-phenotype association was established for *BBS16* (*SDCCAG8*), which is associated with more penetrant early-onset kidney disease and absence of polydactyly/brachydactyly [14]. Furthermore, *BBS17* (*LZTFL1*) is associated with mesoaxial polydactyly, however few patients have been reported [15]. A global trend is to consider relatively milder phenotype for patients with *BBS1* variants, compared with variants in the chaperonin-like genes namely *BBS6*, *BBS10*, *BBS12* [16], as described in a study focusing on the retinal dystrophy [17] or a number of studies focusing on the kidney manifestations and confirmed by a recent meta-analysis [18]. Interestingly, bi-allelic variants in BBS-genes can be associated with non-syndromic isolated Retinitis Pigmentosa [19].

### Clinical diagnosis and monitoring during the life span

#### BBS diagnosis can be made at any age, due to clinical variability of pleiotropic symptoms and asynchronous manifestations

In utero manifestations are often observed during the second trimester prenatal ultrasound (US) examination. However, US is normal in 39% of individuals, with kidney abnormalities or polydactyly detected postnatally [20]. The discovery of bilateral enlarged hyperechogenic kidneys (with or without cystic lesions) should lead to consider BBS as possible diagnosis, especially when concomitant hexadactyly is present. This situation warrants BBS genetic testing. The same stands for hydrometrocolpos.

During childhood and adulthood, diagnosis and management has to be multidisciplinary. Follow-up and care are adapted according to the severity of clinical manifestations and to the general status of the patient (Tables 3 and 4).

**Table 3:** Synopsis for work-up and follow-up of patients with BBS.

FIRST VISIT	
*Family history*	• Ask for: other family members with BBS clinical criteria, BBS typical features or proven BBS testing, parents with metabolic syndrome, obesity or T2D, parental height, other malformation or relevant medical event in the patient’s family including parental consanguinity.
*General personal history*	• Ask for: age at first symptom, presence of situs inversus, surgery for cryptorchidism, removal of accessory digits, symptoms of OSAS, eating behaviour such as hyperphagia.
	• Ophthalmic symptoms: nystagmus, night-blindness, photophobia, bumping into objects, clumsiness and low vision symptoms.
	• Uro-nephrological symptoms: night time drinking & abnormal daily fluid intake, enuresis, secondary enuresis, voiding problems.
	In addition: - Inspection of genitalia and urethra according to endocrinology workout: genital anomalies detection (males: micropenis, testicular position and volume measurement; females: vaginal atresia, hydrometrocolpos …) - Ultrasound kidneys (and urinary tract and internal genitalia if needed)
	• Endocrinology: Target height calculation (in children)
	• Developmental status: Initial morphological examination of the face and body
	In addition: - Search for polydactyly, bradydactyly, scoliosis, urogenital malformations/anomalies… - Any other (rare) associated malformations, situs inversus, cardiac anomalies (US at first visit), constipation…
*Initial work-up & follow-up*	
*Endocrinology*	
*History taking*	• *Eating behaviour • *Symptoms of OSAS • *Physical activity and sedentary time
*Clinical Work-up*	**•** *General status **•** *Pubertal staging
*Growth and statu*s	• *Height, weight, BMI • Adapted: Ambulatory BP 24 h (when blood pressure is above 95th percentile on three separate measurements)
*Blood Test*	• *Fasting lipid profile (TG, LDL, HDL, total cholesterol) • *Fasting glucose and insulin, HbA1c, uric acid • *Liver: AST, ALT, in adult*s*: GGT, blood platelets for calculation of the FIB4 (index of liver fibrosis) • *Thyroid: TSH, freeT4 • IGF1 (in children with growth retardation or on treatment with recombinant GH, in adults) • In minipuberty, teenagers and adults (every 5 years when normal): FSH, LH, total testosterone/estradiol, prolactin, progesterone, SHBG, AMH (females), inhibinB (males) • Following transition to adulthood => Re-evaluation of gonadotroph and GH axis to search for reversibility
*Imaging* *&* *Electro-physiology Testing*	Adapted:
	• Body composition by DEXA (if available) when BMI SDS > 3
	• Abdominal US (for NAFLD) when liver tests are abnormal/hepatomegaly/ hepatomegaly
	• Testicular US in case of testicular ectopy
	• Pelvic US in case of dysmenorrhea, hyperandrogenism
	**•** In adults: - Bioelectrical impedance analysis - Resting ECG if obesity and/or diabetes - Osteodensitometry if hypogonadism - Polysomnography when BMI > 35 or suggestive signs, including questionnaire when BMI SDS > 3
*Question-naires*	Adapted: to age and neurodevelopmental context (presence of intellectual disability in adults): • Eating behaviour questionnaire: SEQ, DYKENS • Sleeping behaviour questionnaire (ESS Epworth sleepiness scale)
Urology-nephrology	
*History taking* *(Each visit)*	*Ask for**:** night time drinking & abnormal daily fluid intake/balance, daily urine output (enuresis, secondary enuresis), voiding problems (frequency, pain, hesitation)
*Clinical work-up* *(Each visit)*	**•** *Presence of oedema, urinary continence attainment - urinary infection (fever) **•** *Ambulatory BP (in adults home BP if feasible), BP 24H*, Height, Weight, BMI **•** *ECG if high BP and/or diabetes
*Blood test*	**•** *Full blood count • *Creatinine, & Estimated glomerular filtration rate with a serum creatinine-based formula adapted to age • *Cystatin C (in case of growth retardation or obesity) • *Urea Nitrogen (BUN) • *Electrolytes: Na + , K + , bicarbonate, Calcium, magnesium, Phosphate, Alkaline phosphatase **•** *25 (OH) vitamin D, PTH
*Urine test*	**•** *Sediment **•** *Creatinine **•** *Protein, albumin (albumin to creatinine ratio to avoid false negatives due to polyuria), micro-albumin
Imaging*	• US kidneys (and urinary tract and internal genitalia if needed) done at first visit is then adapted to initial findings or intercurrent event • Adapted: MRI (teenagers/adults) in case US imaging is too limited due to obesity and technical equipment
* Ophtalmology*	
*History taking*	*Ask for: nystagmus/strabismus, nyctalopia symptoms (not evident for parents), photophobia, loss of visual field symptoms (bumping into objects, clumsiness) & low vision symptoms (loss of daily visual skills, not recognizing people, …)
*Basic ocular examination*	• *Refraction (under cycloplegia for children and young adults) • *Visual Acuity (best corrected) testing with age-appropriated chart • *Slit-lamp (cataract detection in adults)
*Functional testing*	• *Visual field, adapted to age and level of vision
*Imaging*	• *Funduscopy (fundus photograph, if possible wide field or ultra-wide field imaging), • *OCT en face and transfoveal & Fundus Auto Fluorescence
*Electroreti-nogram*	• Adapted: Full field ERG only if needed and usually for initial diagnostic work-up & If needed under general anaesthesia (only for diagnostic value)
*Developmental anomalies*	
Neurological examination	**•** *According to age **•** Adapted: If anomalies: MRI, EEG in case of convulsions
*Neuro-psychological assessment* (accounting for the patient’s visual impairment)	**•** *According to the age and patient: global level of development and search for slowness of ideation, adapted according to the patient’s age and abilities. **•** *Assessment of behavioural problems: emotional immaturity, hyperactivity, intolerance to frustration, disinhibition, obsessive-compulsive disorders, impaired affect **•** *Speech and language assessment
*Orthopaedic assessment*	**•** Adapted: If needed, for polydactyly, bradydactyly, scoliosis
*Craniofacial*	**•** *Dental examination (dental hygiene, crowding, …) **•** Adapted: If needed: Hearing test (Transient Evoked Oto Acoustic Emission test and audiometry) & ENT test to look for conductive or mixed hearing loss

*Yearly follow-up is recommended as baseline (but can be increased due to context)

If any associated condition is detected (CKD, severe obesity, diabetes, etc.) follow-up visits can be increased and adapted to medical field specialty current recommendations for children and for adults.

*Adapted*: means that the item is done only in specific conditions or situations.

Note 1: The main clinical manifestations follow the learned societies and current international guidelines for hypertension in children [59], management of CKD [56], Clinical Practice Guideline for obesity in children [40, 41].

Note 2: *BBS1* mutations with no additional risk factors for CKD, normal BP and no kidney or urinary tract abnormalities on previous examination, this might be reduced to 3 years.

**Table 4:** Synopsis of main points for BBS patient care for BBS main clinical manifestations.

GENETIC COUNSELLING	
*General recommendation*	**•** Genetic counselling is advocated for parents, patients and family members • Genetic molecular testing has to be performed when available
* General anesthesia*	
General recommendation	**•** Detailed and close supervision of any general anaesthesia (GA) with use of advanced procedures (video-laryngoscopy or intubation techniques under bronchial fibroscopy) particularly in adults. • No contraindication to GA if the preoperative, intraoperative and postoperative assessments are closely monitored.
* Endocrinology*	
*Obesity*	• Multidisciplinary care: paediatricians, endocrinologists, dieticians, psychologists, physiotherapist, social workers • Lifestyle recommendations at first: regular physical activity, dietary and eating behaviour follow-up • Pharmacological treatment if accessible and according to indications: - MC4R agonist (Setmelanotide) - Incretin-based pharmacotherapy available for common obesity has to be evaluated in BBS • Obesity surgery**:** bariatric surgery has to be evaluated long-term
*Other endocrine disorders:*	**•** Male hypogonadism: Standard Testosterone replacement therapy (late childhood/adolescence), GnRH pump and gonadotropins can be used in adulthood • Diabetes: Standard Metformin, GLP-1 analogues & SGLT2 inhibitors (NB: these 2 last drugs have not been evaluated) • Hypothyroidism: Standard levothyroxine therapy (dose adapted to age and weight) • Short Stature with proven GH deficiency: Standard recombinant GH in childhood
*Global recommendation*	• Lifestyle measures **(**diet, eating behaviour, physical activity) and psychosocial support are to be considered first for obesity. **•** Setmelanotide and/or incretin-based new therapy should be discussed with expert centres. • Follow current guideline of the International Obesity Task force [40, 41] • Interventions need to be tailored, as regular programs are not suitable for children with developmental delay.
*Uro-nephrology*	
Specific care	**•** Polyuria: Daily management of polyuria and risk of dehydration in case of abnormal fluid losses. • Hypertension: Importance of diet to prevent or treat hypertension, and to prevent or to manage obesity/diabetes [58, 59] • IF CKD=>Follow up is adapted according to current guidelines on management of chronic kidney disease [56]. CKD therapy follow recommendation according to CKD stage: medical, dialysis, transplantation (immunosuppression as recommended / obesity)
Global recommendation	**•** Avoid nephrotoxic drugs in general • If High Blood Pressure=>regular electrocardiogram and cardiac ultrasound are advocated. • If High Blood Pressure & diabetes & CKD=>perform cardiac ischemia test according to recommendation • If dysfunctional voiding=>risk of Urinary Tract Infection to be prevented and managed according to current recommendation
*Ophthalmology*	
Specific care	• Low vision care & visual aids adapted to age • Corrected glasses with tinted filters if needed • School & work adaptation to visual handicap & Rehabilitation sessions • Registration as poorly sighted/blind and follow standard recommendations • White cane/dog/Apps if needed • Cataract surgery if needed • Cystoid macular edema local or systemic therapy if needed
* Developmental anomalies*	
Specific care	• Orthopaedic Surgery for polydactyly (usually in childhood) & scoliosis • Podiatry equipment for brachydactyly • Surgery for urogenital track malformation (ex: hydrometrocolpos) • Neuropsychological or psychiatric care according to evaluation (ranging from occasional care, daily care, to long duration care) • Psychological treatment and family support

Note: they follow the learned societies and current international guidelines referenced herein.

As a general statement, it is important to provide detailed and close supervision of any general anaesthesia (GA) for a patient with BBS, because the use of advanced procedures (video-laryngoscopy or intubation techniques) is often necessary, particularly in adults, as respiratory distress has been observed [21]. There is no contraindication to GA if the preoperative, intraoperative and postoperative assessments are closely monitored.

### Ophthalmic manifestations

#### Ophthalmic clinical synopsis

The prevalence of retinal degeneration (RD), due to degeneration of the photoreceptor cells (cones and rods) is estimated to be > 90% [22, 23]. RD is considered a fully penetrant trait but clinical variability can occur. The spectrum of other BBS systemic manifestations is independent from the retinal phenotype. The mean age for RD diagnosis is between 5 and 12 years of age [23] but the visual symptoms may be obvious before. As not all extra ocular signs are present when RD is diagnosed, this may lead to a delayed diagnosis. Visual impairment in a child with either polydactyly, overweight or a history of prenatal kidney anomaly is suggestive of BBS.

Night blindness is the most common initial symptom in children [23], followed by or together with impaired central vision. Night blindness may be unnoticed by parents. Photophobia may occur with central retinal involvement [24]. Slow adjustment from dark to light environments and vice versa can be observed. Progressive peripheral visual loss, inducing clumsiness, mobility difficulties (bumping into objects, difficulties walking stairs) and ultimately tunnel vision, is common. Often, poor central vision impairs reading or execution of any fine vision tasks. A wide range of refractive errors (myopia, hyperopia, astigmatism) require refractive correction (spectacles) [17]. With age, RD will progress with reduction of visual acuity and visual field worsening.

Most patients are registered as visually impaired (“partially sighted”) or severely visually impaired (“legal blindness”, defined legally as 20 degrees or less of remaining visual field in the best seeing eye, or a visual acuity of 20/200 or worse) by the mid-teens [25, 26]. Strabismus occurs due to the poor vision as well as nystagmus that can be observed early.

Reduced visual function may be present even if the fundus examination is still normal at the early stages. Therefore, recording the function of photoreceptors with an electroretinogram (ERG) is an important exam to confirm RD, especially in young children with limited attention. ERG shows reduced function of rod photoreceptors (scotopic responses) and/or reduced function of cone photoreceptors (photopic conditions) and evolves to usually non-recordable ERG responses (flat ERG) [24].

The RD is usually described as a rod-cone dystrophy (classical Retinitis Pigmentosa) starting with peripheral disease. However a generalised early-onset retinal dystrophy with both central and peripheral features at the time of diagnosis is common due to rapidly evolving RD (rod and cones are affected more or less simultaneously) [17, 24, 26–28]. Early “salt and pepper” appearance can precede to advanced stages with extensive pigment migration [17].

In rare cases, central disease occur with macular features as a starting point (with altered photopic ERGs) with extension towards the periphery (cone-rod dystrophy) or without extension (cone dystrophy) [17].

#### Ophthalmic monitoring and follow-up (Table 3)

Ophthalmic follow-up should be adapted to the age of the patient and cooperation. For visual acuity (VA) evaluation, preferential looking and Teller acuity tests are used for preverbal children, and decimal or Snellen chart in school-age children who disclose often low levels of measurable vision. The VA usually declines over years, leading to severe visual impairment. Rarely, even in the late teens or adulthood, patients can maintain a measurable visual acuity or inversely present preserved visual fields (if central involvement only) [17, 26, 28, 29].

Refraction (under cycloplegia, according to age and recommended guidelines) helps to evaluate the Best Corrected Visual Acuity (BCVA). Regular follow-up of refraction is advocated and full correction should be prescribed unless the visual function is undetectable [30]. Visual field are evaluated with Goldmann kinetic perimetry and performed according to age and remaining vision. Slit lamp evaluation is systematic in adults, as cataract is a frequent complication. Fundus imaging is useful, as it can be captured quite easily and evaluates the various sectors of the retina and the optic disc.

Optical Coherence Tomography (OCT) (if the patient can still fixate) evaluates the degree of outer retinal layers (photoreceptors) alteration and retinal lamination and thickness abnormalities. Autofluorescence imaging assesses the distribution of the RD activity usually with a granular pattern, perimacular ring of hyperautofluorescence.

Full-field ERG is performed mainly if needed for diagnosis. Performing an ERG at young age (or when intellectual disability) may require a GA in case of diagnostic uncertainty.

Clinical follow-up should be performed by a senior ophthalmologist, a low vision specialized orthoptist and/or an optometrist, at least on a yearly basis if the RD is evolving, and every two years if the situation is stable in adults.

#### Ophthalmic management (Table 4)

No specific therapy for RD in BBS patients is available to date. A few patients have benefited from retinal implants at the late stage of RD for trial purposes. Gene therapies, optogenetics, and cell replacement may be future avenues. Currently, patients benefit from low visions aids, electronic apps, orientation and mobility training, Braille, white canes and guide-dogs. Specific training for poor vision is adapted to the age. Technological/electronic assistance is commonly used, except if hampered by intellectual disability. Refractive errors are observed in 90% of cases and should be corrected. Tinted glasses with photoselective filters may be useful in case of photophobia.

Cataract is a common complication of RD usually in early adulthood [17]. Surgery with intraocular lenses implantation is advocated when the dense opacities are central and aggravate the visual function. Cystoid macular oedema, a classical complication of RD, is rare and should follow the classical therapy.

#### Ophthalmic findings for phenotype genotype correlations

Intrafamilial variability has been observed in the onset and course of disease [31]. Central and peripheral visual dysfunction can be variable between patients carrying pathogenic variants in the same genes. Rare prominent central retinal diseases with relative rod sparring has been associated to various genes (*BBS1, BBS10, BBS6, BBS5* and *BBS12)* [29]. Some patients with non-syndromic RD (no extra ocular features detected) can carry bi-allelic variants in BBS genes: *BBS1* [32], *BBS2* [19], *BBS7* [29]*, BBS8* [33], *BBS10* [17] *IFT172* [34], *C8orf37* [35]. Patients with pathogenic variants in *BBS1* seem less severely visually affected compared to those carrying variants in other genes (*BBS2*, *BBS4*, *BBS10*) [17, 36]. However, *BBS1* gene is subject to extreme phenotypic variability with very mild long-term expression but sometimes present with typical early onset RD [37].

### Obesity and endocrine disorders

#### Obesity and growth clinical synopsis

During childhood, height assessments disclose variations ranging from short stature ( < 3rd percentile) to a stature above normal range (at the 90th percentile). However, most patients grow up below the median (25–50th percentile). During adolescence, height returns to the normal range. Final height on average is similar or slightly lower than the general population [1].

Almost all patients develop obesity at some point. Despite normal birth weight, most individuals experience rapid weight gain in early childhood (before 5 years old) with weight z-scores above 2.0. In children > 5 years, 90% disclose overweight or obesity maintained through adolescence. In adults, the obesity prevalence rate is very high (74–100%). About 25% of patients are expected to develop grade 3 obesity as adults (defined by a Body Mass Index (BMI) > 40 kg/m^2^) [2, 10, 38]. Eating disorders, including hyperphagia and food-seeking behaviour are reported in most children and adolescents. Hyperphagia has been observed since early in life but data is still lacking for the life span. Obstructive sleep apnea and sleep disturbances (OSAS) are extremely common and linked to BMI [39]. An increased prevalence of cardiovascular risk and thromboembolic events has been noted.

#### Metabolic BBS-related disorders clinical synopsis

Prevalence of metabolic disorders (insulin resistance and glucose intolerance, metabolic syndrome and hypertriglyceridemia, Non-alcoholic Fatty Liver Disease (NAFLD) is higher in BBS adults compared to other obese patients [40]. Hypertension, insulin resistance and type 2 diabetes are more frequent and present at younger age in BBS patients [2, 40]. At birth, no symptoms of metabolic disorders are observed, however, during the first and second decade of life, symptoms can appear and progressively worsen.

#### Endocrine disorders clinical synopsis

Hypogonadism in males is the most frequently reported endocrine disorder (20–80%). From birth, BBS boys can present with genital variations, such as micropenis, cryptorchidism and small testes evocative of congenital hypogonadotropic hypogonadism. Delayed puberty is described as well as some cases of precocious puberty. In adult males with hypogonadism, it is mostly of central origin (85%), but primary hypogonadism is also documented. Results from a recent study involving 11 BBS male patients suggest that primary cilia dysfunction in BBS affects the embryology of the male genital tract, in particular the epididymis, but spermatozoa structure in adults does not appear to be impacted. These findings need to be confirmed by studies on larger cohorts of BBS patients, focusing on fertility. Some male subjects had fathered children [41].

Females can present with hydrometrocolpos and/or vaginal atresia. Puberty is generally normal in girls but around 15% of BBS women present a polycystic ovary syndrome. Fertility seems to be preserved in women, pregnancies have been reported, but primary ovarian failure has been documented [10].

Growth hormone (GH) deficiency is rare, however MRI structural pituitary abnormalities (hypoplastic pituitary, empty sella, Rathke cleft cyst, olfactory bulb aplasia) seem to be frequent (with or without pituitary hormonal impairment). Cases of hyperprolactinemia have been described, although there is no available data on prevalence. In adults, thyroid dysfunction due to primary hypothyroidism (subclinical or overt) is reported at a rate of 19.4%. No case of central hypothyroidism has been described so far. No case of corticotropic insufficiency was reported, however blindness can disrupt cortisol circadian rhythm. Autoimmune diseases such as type 1 diabetes and Hashimoto thyroiditis have been described.

#### Obesity and endocrine management (Tables 3 and 4)

Given the complex nature of early-onset severe obesity, treatment should ideally be provided by a multidisciplinary team with: medical providers, registered dietitians, psychologists, physical therapists, and social workers.

Obesity in BBS patients is responsive to calorie restriction. Combined Lifestyle modification therapy, including behaviour modifications such as family involvement and education on dietary and physical activity is the first step. Treatment options of obesity-related disorders do not change from the usual standards of care [42, 43]. Regular and sustained physical activity and breaks in sedentary time should be encouraged as a way to improve metabolic comorbidities. Physical activity should be adapted to visual deficiencies and neurodevelopmental status.

For BBS patients with diabetes, treatments targeting insulin resistance without causing weight gain have to be prioritized (metformin, incretin-based treatments, SGLT2 inhibitors). BBS patients with obesity and/or hyperphagia can be eligible for treatment by MC4R agonists. Setmelanotide is currently approved by the European Medicines Agency for children aged six and above and is reimbursed in a number of EU countries [44]. This prescription should be discussed by BBS expert centres. New pharmacotherapies, such as incretin-based treatments (i.e semaglutide, tirzepatide) appear in the therapeutic arsenal for common obesity, but the specific effects in BBS patients have yet to be evaluated [45].

While studies show cases where bariatric surgery had rather encouraging results for rare genetic obesity disorders (including BBS) that are comparable to common polygenic obesity, the studies have only short and medium term follow-up. There is limited high-quality evidence to support bariatric surgery as a treatment option for rare genetic obesity disorders on the long term [46, 47]. Surgery may be considered with caution in cases of BBS with neuropsychological disorders (intellectual deficiency and/or behavioural disorders) and should be discussed with an expert team. The benefit-risk balance should be taken into account: 1) the long-term effects on weight; 2) the inherent risk of multi-organ damage linked to the syndrome (digestive, respiratory, anaemic and thromboembolic risk); 3) the psychological vulnerability and eating behaviour disorders that represent exclusion factors for bariatric surgery; 4) increased risks in both GA and postoperative complications that can be masked due to difficulties in feeling discomfort and expressing complaints.

#### Endocrine disorders management (Table 3)

Treatment of central/secondary or primary hypogonadism is not specific to BBS and modalities are the same than the general population guidelines. Testosterone replacement therapy is a well-tolerated and established treatment, providing excellent clinical relief and biochemical efficiency for sex steroid deficiency. In adults, fertility desire can modulate therapeutic options (use of GnRH pump, gonadotropins). In patients with behavioural issues, androgen therapy has to be monitored with caution. As reversible hypogonadism was reported, revaluation of the gonadotropic axis in adulthood (after withdrawal of androgen therapy) has to be considered.

Treatment of diabetes/dyslipidaemia/NAFLD is not specific to BBS and modalities are the same than in common obesity [42, 43].

In case of hypothyroidism, a replacement therapy with L-thyroxin should be introduced with modalities similar to the general population.

In case of growth hormone deficiency or severe chronic kidney disease, treatment with recombinant GH is indicated in children.

#### Endocrine and metabolic clinical monitoring and management (Table 3)

Assessment by a professional with expertise in eating disorders is essential. The standard curve for the considered population and International Obesity Task Force criteria to define overweight and obesity should be used to diagnose obesity/overweight, while taking into account that the weight-related health risk is already increased by BBS itself. Cases of endometrial cancers possibly related to obesity have been described. Hormonal and metabolic parameters should be monitored according to the guidelines of the general population with obesity [42, 43]. A yearly follow-up, monitoring the weight, eating disorders, obesity comorbidities, and including a metabolic assessment should be implemented for both paediatric and adult patients. If an issue arises, monitoring should be increased accordingly, following usual recommendations.

In children and adolescents, ratings of eating behaviour can be provided by parents range from 1 (no obvious eating issues) to 5 (important eating issues and foraging). The hyperphagia questionnaire by Dykens used for Prader-Willi Syndrome is useful to diagnose and evaluate the severity of eating disorders in children and adolescents, especially for the food-seeking activities [48]. By extension, in adults with intellectual deficiency (ID), questioning the caregiver and/or parents with the same tool can be useful. In children or adults with ID and/or autism, the significant event questionnaire (SEQ) is a weekly eight-item novel instrument, care-giver reported outcome measure designed to capture rare food-related behaviours (i.e. of interest especially for treatment response) [49]. Total scores range from 0 to 24, with higher scores suggestive of more significant appetite suppression. In adolescents or adults, assessment of their hunger levels and eating behaviours using face-to-face interview is recommended. Other validated questionnaires can also be used. None of the questionnaires have been validated in BBS specifically. All BBS patients should be routinely screened for symptoms of OSAS (Polysomnography).

#### Phenotype genotype correlations findings for obesity and endocrine manifestations

Despite the lack of proper statistical evidences, some phenotype genotype trends have been highlighted. *BBS1* seems associated to a milder endocrine and metabolic phenotype than all other BBS gene defects. *BBS1* comorbidities were found as frequent as in obese controls, while in other genotypes the comorbidities frequencies were found increased. Obesity and hypogonadism in *BBS1* are less frequent than in patients with *BBS2* or *BBS10*. They also present lower levels of insulin resistance (lower HOMA-IR) and lower visceral adiposity than *BBS10* patients, who have a trend toward increased insulin resistance (higher HOMA-IR) and increased triglycerides. *BBS4* was linked to the greatest BMI difference compared to non-BBS patients [1]. Recent association studies have suggested *BBS9* to be associated with hyperglycaemia and insulin resistance [50]. With respect to height, on average, *BBS1* patients are taller and *BBS2* and *BBS4* patients are shorter than their target height during childhood, compared to other genotypes. For reproductive disorders, no consistent phenotype-genotype correlation was found.

### Kidney and urinary tract involvement

All patients with BBS should undergo a nephrological evaluation to detect underlying abnormalities of the kidneys and urinary tract, and should benefit from ad hoc follow-up monitoring. Overall, the frequency of kidney and urinary tract disorders occurs in over 50% of patients.

#### Kidney and urinary tract disease clinical synopsis

There are no specific urinary tract disorders associated with BBS, and the incidence varies.

Structural abnormalities of the kidneys and urinary tract comprise: persistent foetal lobulation, increased echogenicity of the kidney parenchyma, loss of corticomedullary differentiation, cystic disorders, kidney hypo- or dysplasia, dilatation of the upper urinary tract, vesico-ureteral reflux, duplex systems and horseshoe kidneys. Patients with underlying severe kidney and urinary tract development abnormalities are at risk to develop Chronic Kidney Disease (CKD).

CKD is an important but not an obligatory feature in BBS. The uro-nephrologic phenotypic spectrum is non-specific, ranging from isolated urinary tract disorders to severe kidney dysplasia in association with macro- and microcystic lesions. Patients with kidney involvement often present a reduced urinary concentrating capacity manifesting with polyuria/polydipsia. Reduced urinary concentrating capacity indicates an underlying CKD, implying the risk of progressive Glomerular Filtration Rate decline [51]. High blood pressure (BP) is frequent with a prevalence increasing with age [52–54].

Although CKD is frequent in patients with BBS, only a minority develops kidney failure (KF) with the need for renal replacement therapy. KF may occur at any age. However, among paediatric patients with CKD, KF has been shown to peak at the first year of life [53]. In the largest cohort study to date, KF occurred in 6% of adult and 5% of paediatric patients with BBS, respectively [53]. Adult-onset severe CKD may also relate to comorbidities (obesity, diabetes, urinary tract infections and hypertension). KF prevalence was higher in females than in males in recent series [55–57]. Voiding dysfunction is quite common in BBS patients (25%) and may cause recurrent urinary tract infections.

#### Management of kidney and urinary tract disease (Tables 3 and 4)

Management of kidney and urinary tract disease in BBS is not different from other causes and should be in accordance with common current guidelines [58, 59]. Urological manifestations may require specialist management. Medical work-up regarding kidney complications is similar in paediatric and adult patients (Table 3). Blood pressure (BP) should be assessed and treated according to current recommendations in children and adults with CKD [60, 61]. Where feasible, ambulatory BP measurement should be included in the follow-up of patients with BBS with hypertension, CKD or obesity. The dietary recommendations for patients with BBS and CKD should further include the current age-appropriate guidelines on diet for patients with CKD.

In case of KF, there is no evidence that patients with BBS should not be a candidate for kidney transplantation (KT), as outcomes are comparable to those of the general population. However, severe obesity can be a relative contra-indication to KT. Inappropriate increase of the median BMI post-transplant in patients with BBS has been reported, thus steroid sparing regimen might be considered [62]. Evaluation of cardiovascular abnormalities [63, 64] should be performed before KT. Adherence to immunosuppressive medication after transplantation must be evaluated according to the level of intellectual disability and family support. The risk of New-Onset Diabetes after Renal Transplantation (NODAT) is increased in BBS due to obesity, which should be taken into account in the choice of the immunosuppressive regimen, in particular with the use of tacrolimus.

#### Phenotype-genotype findings for kidney diseases

Truncating variants in any genes or mutations in chaperonin-like genes correlate with a higher risk to develop CKD [18]. Comparing the two most common loci in the western countries, patients with *BBS1* mutations are more likely to have either no CKD or mild to moderate CKD, whereas patients with mutations in *BBS10* are likely to have severe CKD [53]. Among BBSome components, kidney anomalies showed a low frequency in patients with mutations in *BBS1, BBS4*, or *BBS8* and a high frequency in those with mutations in *BBS2, BBS7*, or *BBS9*. Patients harbouring variants in chaperonins (*BBS10*, *BBS12* and *BBS6)* are more likely to develop severe CKD than patients with *BBS1* mutations. *BBS3* (*ARL6*) deficiency was characterized by a lower penetrance of kidney anomalies as well as *BBS22* (*IFT174*) [65].

### Developmental anomalies

#### Developmental anomalies clinical synopsis

Developmental anomalies (DA) are heterogeneous but frequent (however large series are lacking for robust incidence). Major malformations can be detected in utero or at birth. The main DA is polydactyly, a major feature (60–80%) occurring with variable locations for upper and/or lower limbs [66]. In most cases, polydactyly is postaxial and exceptionally mesoaxial (*BBS17*). Brachydactyly is a common feature and may occur alone.

Urogenital track DA are common (50% [66] and warrant early detection with systematic US at birth. In female patients, malformations can include hydrometrocolpos (in extreme forms, presenting as an abdominal tumour), hypoplastic fallopian tubes, uterus, or ovaries, vaginal atresia or vesicovaginal fistula. In male patients, the malformations may include cryptorchidism or small penis length [2].

Facial gestalt particularities have been noted but are not useful for diagnosis, as they have never been studied systematically. Dental anomalies have been reported in over 50% of patients with BBS [67], manifesting as hypodontia, dental crowding or high-arched palate, but are of little value for BBS diagnosis. However, oral care is important for the individual’s general health and should not be missed. Congenital heart defects occur in about 7% of cases [2, 10, 66], and care follows standard procedures as for the general population. Heterotaxia with situs inversus and abnormal central veins (bilateral persistent superior vena cava, interrupted inferior vena cava, and hemiazygos continuation) occur in less than 1.6% of cases [68].

Gastrointestinal conditions, such as Hirschsprung disease, have not been evaluated for their incidence but have been noted in a few case reports. Liver structures anomalies were reported in 26.22%, and around 30% of patients with BBS present with liver disease [3] that are mainly linked to obesity. Patients with BBS have higher rates of musculoskeletal and orthopaedic problems. Joint laxity (20–28%), scoliosis (16%) are the most common issues [3].

Neurodevelopmental conditions are common, heterogeneous and sometimes complex. Cognitive impairment, learning disability, behavioural dysfunction are reported to be around 66% [66], but systematic data is lacking. Approximately 20–25% of patients meet criteria for intellectual disability (ID) [69], but the literature is not always clear on the distinction between ID and learning disability, which is found in 60% of patients [10, 66].

The following traits have been noted: mean intellectual functioning at − 1.5 SD below the mean, –impairments in verbal fluency (22–44%) and perceptual reasoning (53%), reduction in attention capacity (69%) and lack of functional independence (74%) [69], though most patients do not have ID [69, 70]. Autism spectrum disease (ASD), presenting as behavioural rigidity, sensory sensitivity, issues in peer socialisation or social and/or emotional reciprocity, have been found to occur in 77% of patients [3, 69]. Behaviour and psychiatric features occur in around 30% of patients, with obsessive-compulsive behaviours, anxiety and mood disorders [10].

Receptive and expressive speech delay and hypernasal speech have been observed and are in part linked to hearing problems, as mild hearing deficiency is found in 17–21% of patients [66].

Impaired learning and memory could be associated with pathological alterations in hippocampal primary neuronal cilia. On morphological grounds, MRIs scans of the CNS have identified hippocampal dysgenesis with reduced hippocampal volume, hypoplasia of olfactory bulbs, cerebellar hypoplasia and cerebellar atrophy [71].

Anosmia is common and thought to be linked to hypoplasia of olfactory bulbs [72].

Epilepsy has been reported (4–10%) [2, 3, 10], and so has ataxia, with impaired motor coordination, but is poorly documented [10, 66].

#### Management of developmental anomalies (Tables 3 and 4)

Overall, DA management do not usually differ from the recommended care in patients without BBS. Early cardiac and abdominal-genital US are recommended, and any pathological findings have been managed according to current recommendations. Urogenital track malformations can require neonatal surgery. In females, gynaecologic follow-up is advocated.

Surgery for polydactyly can be advocated either at birth (post-minimus appendage) or in early infancy for non-functional fingers or toes. Orthopaedic equipment is often warranted for lower limb care, as shoe-fitting may be problematic due to the brachydactyly and enlarged short feet.

Neuropsychological or psychiatric evaluation may be necessary and adapted to the workup. Early detection of ID, learning deficit and ASD imply remediation following national specificities and should anticipate visual degradation.

Psychological follow-up is often advocated during the life span. Initially, psychological treatment may be useful to detect any behavioural problems, or to provide treatment depending on the symptoms presented, but it can also help older patients learn to live with a progressive and chronic disease that can lead to severe handicaps and optimise social and professional integration.

#### Main differential diagnosis

Differential diagnoses will depend on the clinical presentation and the patient’s age. They can range from single-organ diseases to syndromes linked mainly to ciliopathies when the clinical spectrum overlap with BBS symptoms [22]. Genetic testing with panels including genes for ciliopathies is crucial in order to identify the diagnosis precisely in all cases cited hereafter (See supplementary data).

Early-onset retinal degeneration, especially if obesity is present, should prompt for BBS genetic screening. Alström Syndrome patients, one of the main differential diagnosis, also present with early-onset retinopathy (but with earlier nystagmus and intense photophobia), no polydactyly, obesity and may also progress to renal failure. The occurrence of transient cardiomyopathy in early life (and often recurring later) is highly suggestive of ALMS as well as early onset of insulin resistance and progressive sensorial deafness. Only one gene (*ALMS1*, OMIM*606844) is known and has to be tested on the panels.

## Conclusions

Announcing BBS diagnosis must be the subject of a dedicated consultation and should include an explanation of the diagnosis, information about the natural history and prognosis, the need for regular monitoring and the scheduling of examinations to monitor the disease and detect complications, and, when applicable, the treatments prescribed with possible side effects descriptions. A planning of treatment and follow-up, together with a description of the multidisciplinary teams. Genetic counselling for the patient and family should also be covered. Psychological support for affected individual and family is of high importance at diagnosis and follow-up whenever needed.

Genetic counselling is essential, as early as possible, in order to explain to patients and families the disease and the importance of determining the inheritance pattern. Genetic advice can be given regarding the risk of recurrence in case of pregnancy.

Therapeutic education aims to ensure the understanding and involvement of the person with BBS and their family and friends, in order to help patients and their caregivers acquire the skills necessary to manage their lives as effectively as possible. Specialized care in reference centres is advocated and requires specific attention for adults as the patients are often dependant for visits. Therapeutic education will focus mainly on nutritional education, nephrology clinical monitoring and teaching people how to use vision rehabilitation equipment.

Transition from childhood to adulthood has to be tightly monitored with all medical fields. The same stands for transition from adulthood to old age. Associations play an active role in therapeutic education by informing, guiding, helping and supporting patients and their families. Patients’ associations available should be presented to the patient and their families during the announcement of the diagnosis but the choice to contact them is left to the patient.

BBS is recognised as a classical syndromic cause of obesity, retinal dystrophy and CKD. As a ciliopathy, the care has to be multidisciplinary and launched as soon as the diagnosis is ascertained. The genetic testing, with now routine access to NGS technologies, enables quicker and easier diagnosis and genetic counselling. Genetic testing has been added in the novel BBS diagnosis criteria proposed herein. Preclinical research will hopefully lead in the coming years to a better understanding of the condition pathogenesis and to more specific therapies.

## Supplementary information

Table for BBS Differential Diagnosis
