# Supplementary material for: Bardet-Biedl syndrome improved diagnosis criteria and management: Inter European Reference Networks consensus statement and recommendations
Source: Eur J Hum Genet. 2024 Jul 31;32(11):1347–60. doi: 10.1038/s41431-024-01634-7 (PMC11576898; doi:10.1038/s41431-024-01634-7)
Supplement: Supplementary file 1 — Table for BBS Differential Diagnosis [file 41431_2024_1634_MOESM1_ESM.docx]

|  | **Genes Names & OMIM numbers** | **Main OMIM clinical entries**  **(PS = phenotypic series)** | **ORPHA code** | **Inheritance** | **Main Age at Differential Diagnosis** | **Overlapping feature (s)** | **Main difference(s)** | **Comment** |
| --- | --- | --- | --- | --- | --- | --- | --- | --- |
| Albright hereditary osteodystrophy spectrum | *GNAS* (*139320) locus (inactivating variants or methylation anomaly) | #103580  #603233  #612463 | 79443  79444  79445 | AD | EC, AL | OB, ID, SX | - PTH resistance - Hypocalcemia - Other endocrine anomalies (TSH resistance, GH deficiency…) - Subcutaneous ossifications - Brachydactyly | - Several variants (PHP1a, PHP1b, PPHP), based on molecular mechanism. |
| Alström syndrome | *ALMS1* (*606844) | #203800 | 64 | AR | EC, AL | OB, KF, RD | - Early dilated cardiomyopathy - Liver steatosis - Insulin resistance with type 2 diabetes - Progressive deafness |  |
| Carpenter syndrome | *RAB23* (*606144)  *MEGF8* (*604267) | #201000  #614976 | 65759 | AR | AL | PD, OB, SX | - Craniosynostosis - Polysyndactyly - CHD |  |
| Cohen syndrome | *VPS13B* (*607817) | #216550 | 193 | AR | AL | OB, RD, ID | - Distinctive facial dysmorphism - Myopia - Cyclic neutropenia |  |
| Joubert syndrome | *CEP104* (*616690)  *NPHP1* (*607100)  *TMEM237* (*614423)  *ARMC9* (*617612)  *PDE6D* (*602676)  *ARL13B* (*608922)  *CC2D2A* (*612013)  *CPLANE1* (*614571)  *CEP120* (*613446)  *AHI1* (*608894)  *CEP41* (*610523)  *CSPP1* (*611654)  *TMEM67* (*609884)  *IFT74* (*608040)  *INPP5E* (*613037)  *FAM149B1* (*618413)  *TCTN3* (*613847)  *SUFU* (*607035)  *ARL3* (*604695)  *TMEM138* (*614459)  *TMEM216* (*613277)  *TMEM218* (*619285)  *CEP290* (*610142)  *TECT1* (*609863)  *TCTN2* (*613846)  *PIBF1* (*607532)  *TOGARAM1* (*617618)  *KIAA0586* (*610178)  *KIF7* (*611254)  *KATNIP* (*616650)  *ZNF423* (*604557)  *ZNF423* (*604557)  *RPGRIP1L* (*610937)  *TMEM231* (*614949)  *KIAA0753* (*617112)  *TMEM107* (*616183)  *B9D1* (*614144)  *MKS1* (*609883)  *B9D2* (*611951)  *OFD1* (*300170) | PS213300 | 140874 (group of disorders) | AR | PN, AL | KF, RD, PD | - Ataxia - Cerebellar vermis hypoplasia - Molar tooth sign - Oculomotor apraxia |  |
| Laurence-Moon syndrome | *PNPLA6* (*603197) | #245800 | 2377 | AR | AL | OB, RD | - Ataxia - Peripheral neuropathy - Spastic paraplegia | - A neurological condition merged with BBS in early nosology - Allelic with Oliver-McFerlane syndrome (with trichomegaly) |
| Leptin deficiency and leptin receptor deficiency | *LEP* (*164160)  *LEPR* (*601007) | #614962  #614963 | 66628  179494 | AD | EC, AL | OB |  | - Non syndromic, early onset morbid obesity |
| McKusick Kaufman syndrome | *MKKS/BBS6* (*[604896](https://www.omim.org/entry/604896)) | #236700 | 2473 | AR | AL | HC, SX  Hydrometrocolpos |  | - Allelic to BBS6 |
| Meckel-Gruber syndrome | *KIF14* (*611279)  *NPHP3* (*608002  *CC2D2A* (*612013  *TXNDC15* (*617778  *TMEM67* (*609884  *TMEM216* (*613277  *CEP290* (*610142  *TCTN2* (*613846  *RPGRIP1L* (*610937  *TMEM231* (*614949  *TMEM107* (*616183  *B9D1* (*614144  *MKS* (*609883  *B9D2* (*611951) | PS249000 | 564 | AR | PN | LK, PD, SX | - Lethal pre- or perinatally. - Occipital encephalocele - CNS anomalies - Cystic kidneys | - The phenotypes corresponds to the severe end of BBS spectrum |
| Orofaciodigital syndromes | *DDX59* (*615464)  *IFT57* (*606621)  *INTU* (*610621)  *CPLANE1* (*614571)  *TCTN3* (*613847)  *C2CD3* (*615944)  *KIAA0753* (*617112)  *TMEM107* (*616183)  *OFD1* (*300170)  *OFD8* (*300484)  *OFD10* (*165590)  *OFD2* (*252100)  *OFD3* (*258850)  *OFD9* (*258865)  *OFD7* (*608518)  *OFD11* (*612913) | PS311200 | 140997 (Group of disorders) | AR, XL | PN, EC | LK, PD (in OFD1), RD | - Lingual hamartoma - Tibial hypoplasia - Agenesis of the corpus callosum | - Group of ciliopathies overlapping with BBS and SRTD |
| Polycystic kidney disease 1, with or without polycystic liver disease | *DZIP1L* (*617570)  *DNAJB11* (*611341)  *PKD2* (*173910)  *FCYT* (*606702)  *GANAB* (*104160)  *ALG5 (**604565)  *PKD1* (*601313) | PS173900 | 730 (AD)  731 (AR) | AD, AR | PN | LK | - Prenatal US isolated feature |  |
| Prader-Willi syndrome | Anomalies in 15q11-q13 region encompassing *SNRPN* gene | #176270 | 739 | Complex | EC, AL | OB (later onset), ID | - Severe neonatal hypotonia - Failure to thrive in early infancy | - Paternally derived deletion, maternal disomy or imprinting defects |
| Senior-Løken syndrome | *NPHP4* (*607215)  *SDCCAG8* (*613524)  *NPHP1* (*607100)  *TRAF3IP1* (*607380)  *IQCB1* (*609237)  *SLSN3* (*606995)  *WDR19* (*608151)  *CEP290* (*610142) | PS266900 | 3156 | AR | EC, AL | KF, SK, RD | - Congenital amaurosis |  |
| Cranioectodermal syndrome (Sensenbrenner) | *IFT122* (*613602)  *WDR35* (*606045)  *IFT43* (*608151)  *WDR19* (*614068) | PS218330 | 1515 | AR | EC, AL | SK, RD KF | - Ectodermal anomalies - Hepatic fibrosis |  |
| Short Rib-Thoracic Dysplasia (SRTD) spectrum | *WDR35* (*613602)  *IFT172* (*607386)  *DYNC2LI1* (*617083)  *TTC21B* (*612014)  *IFT80* (*611177)  *DYNLT2B* (*617353)  *EVC2* (*607261)  *EVC* (*604831)  *WDR19* (*608151)  *INTU* (*610621)  *NEK1* (*604588)  *CEP120* (*613446)  *WDR60* (*615462)  *WDR34* (*613363)  *DYNC2H1* (*603297)  *IFT81* (*605489)  *KIAA0586* (*610178)  *IFT43* (*614068)  *SRTD1* (*208500)  *IFT140* (*614620)  *KIAA0753* (*617112)  *IFT52* (*617094)  *SRTD12* (*269860) | PS208500 | 93426 (Group of disorders) | AR | PN | PD, prenatal echographic signs | - Thoracic bone dysplasia - Early lethality in some | - Group of AR ciliopathies with narrow thoracic cage, short tubular bones, abnormal pelvis, and variable PN liver and kidney involvement. - SRTD, encompasses e.a.Ellis-van Creveld, Jeune syndrome, short rib-polydactyly, and Mainzer-Saldino syndromes. |
| Simpson - Golabi -Behmel syndrome | *GPC3* (*300037) | #312870 |  | XL | PN | LK, PD, ID, SX | - Pre- and postnatal overgrowth - Diaphragmatic hernia - Dystinctive dysmorphism | - Increased tumour risk |
| WAGR and WAGRO syndrome | 11p13 deletion:  *WT1* (*607102)  *PAX6* (*607108)  Sometimes *BDNF* (*113505) | #194072  #612429 | 893 | AD | AL | OB (WAGRO), KD, ID, SX | - Aniridia - Glaucoma - Wilms’ tumor | - Contiguous gene syndrome |

**Legend:**

AD: Autosomal Dominant; AL: All Life; AR: Autosomal Recessive; CHD: Congenital Heart Defect; EC: Early Childhood; ID: Intellectual Deficiency/learning disability; KF: Kidney Failure; LK: Large Kidneys; OB: Infantile-onset Obesity; PD: Polydactyly; PN: Prenatal; RD: Retinal Degeneration; SK: Skeletal Anomalies; SX: Hypogonadism and other anomalies of sexual development; XL: X-linked.
